# Molecular Dynamics Investigation of the Diffusion Mechanisms and Thermodynamic Behaviors in Warm Mix Recycled Asphalt Binders with and Without Rejuvenators

**DOI:** 10.3390/ma18030703

**Published:** 2025-02-05

**Authors:** Qisheng Hu, Derun Zhang, Peixin Xu

**Affiliations:** 1 School of Civil Engineering and Architecture, Wuhan Polytechnic University, Wuhan 430023, China; 2 School of Civil and Hydraulic Engineering, Huazhong University of Science and Technology, Wuhan 430074, China; derunzhang@hust.edu.cn

**Keywords:** asphalt pavement, warm mix asphalt, rejuvenator, molecular dynamics, simulations

## Abstract

In recent years, the employment of rejuvenators and warm mix asphalt (WMA) additives for reclaimed asphalt pavement (RAP) has been recognized as a popular approach to increase the recycling rate of waste materials and promote the sustainable development of pavement engineering. However, the composition of warm mix recycled asphalt binder is complicated, and the microstructural changes brought about by the rejuvenators and WMA additives are critical in determining its macroscopic mechanical properties. This research focuses on the atomic modeling of the rejuvenators and WMA additives diffusion behavior of the warm mix recycled asphalt binder. The objective is to reveal the thermodynamic performance and diffusion mechanism of the WMA binder under the dual presence of rejuvenators and WMA additives. Three types of mutual diffusion systems (Aged and oil + virgin + wax, Aged + virgin + wax, and Aged and oil + virgin) were established, respectively, for a comparative investigation of the glass transition temperature, viscosity, thermodynamics, free volume, and diffusion behavior. The results indicate a 44.27% and 31.33% decrease in the glass transition temperature and apparent viscosity, respectively, after the incorporation of 5% oil rejuvenators in the Aged + virgin + wax asphalt binder, demonstrating the improved cracking resistance and construction workability. The presence of the RAP binder and organic WMA additives raised the cohesion of the asphalt binder and decreased self-healing ability and free volume, and these detrimental influences can be offset by the introduction of rejuvenators. The combined use of rejuvenators and organic WMA additives remarkably enhanced the de-agglomeration to asphaltenes, stimulated the activity of aged RAP macromolecular components, and ultimately improved the blending efficiency of virgin binders with the overall structure of RAP binders.

## 1. Introduction

Due to long-term exposure to complex natural environments and heavy traffic loads, numerous literature studies have shown that the performance of asphalt pavements decays with the aging of the asphalt binder [1,2,3]. At the molecular level, the oxidation of the asphalt binder by external factors leads to the creation of carbonyl and sulfoxide functional groups within the asphalt, tremendously increasing the polar substances in the asphalt binder [4]. Meanwhile, aging changes the SARA four components (saturate, asphaltene, resin, and aromatic) of the asphalt binder [5]. The light components, i.e., aromatic, evaporate as aging processes, while the proportion of macromolecules (asphaltenes) increases remarkably [6]. These microscopic molecular structure changes accompanying the chemical reactions in aged asphalt binders are critical to the degradation of their mechanical properties [7]. At the macroscopic scale, the hardness and brittleness of aged asphalt pavements increase, leading to a decrease in ductility [8,9]. While aging enhances high-temperature rutting resistance in asphalt pavements, it significantly increases the risk of low-temperature and fatigue cracking [10]. According to estimates in China, about 790 million tons of old asphalt pavement are generated annually due to aging. Recycling and reusing old asphalt pavement, generally named reclaimed asphalt pavement (RAP), offers significant economic and environmental advantages [11,12]. One common approach to improve the performance of RAP is pre-blending rejuvenators with RAP materials [13]. However, the widely used hot mix recycling technology has a construction temperature as high as 160–180 °C, putting the RAP at risk of secondary deterioration [14]. Therefore, in practical engineering applications, the amount of RAP incorporated into new asphalt pavements is typically restricted to under 30% [15].

Warm mix asphalt (WMA) technology involves incorporating an appropriate quantity of WMA additives into asphalt or its blends to reduce the construction temperature [16]. Referring to existing studies, it can be found that the reduction in construction temperature by utilizing WMA technology ranges from about 20 to 40 °C [17]. Compared to the production of hot mix asphalt (HMA), WMA significantly reduces both fossil energy use and asphalt fume emissions [18]. However, the reduced production and compaction temperatures result in weaker bonding between asphalt and aggregate in WMA, elevating the risk of high-temperature rutting deformation [19]. To enhance the sustainable advancement of asphalt pavements, researchers have suggested integrating RAP recycling with WMA technology to offset the drawbacks of both methods [20,21,22]. With the reduction in construction temperature, WMA technology can raise the RAP content to 60% or higher [23]. Numerous literature reviews have also pointed out that WMA-RAP technology is the main research direction for current and future green asphalt pavements [24,25,26].

The performance of warm mix recycled asphalt binder is critical to ensuring the long-term durability of the regeneration asphalt pavement [27]. Xu et al. examined the rheological–physical–chemical characteristics of 48 types of warm mix recycled asphalt mastic with a high RAP binder content [28]. Based on rheological performance tests, Ameri et al. explored the effect of different RAP content on the viscosity, high-temperature creep recovery, and fatigue cracking properties of warm mix recycled asphalt binders [20]. Wang et al. studied the effect of two warm mix additives and three RAP contents on the glassy transformation behavior of asphalt binders [29]. These studies improve the ability to explain the mechanism of warm mix regeneration at the macroscopic scale, providing reasonable suggestions for scholars to develop WMA-RAP technology. However, some scholars believe that the introduction of rejuvenators in WMA-RAP technology is only necessary when the RAP dosage exceeds 50% to control construction costs [30]. Other scholars suggest that rejuvenators are necessary, even at low RAP content, to enhance both fatigue and moisture resistance in regenerated asphalt pavements [31,32]. The composition of warm mix recycled asphalt binder is complicated, consisting of RAP binders, virgin binders, WMA additives, and rejuvenators [33]. The microstructural changes brought about by the interactions between the complex components are the hinge to determining the macro mechanical properties of warm mix recycled asphalt binders [34]. Hence, it is imperative to reveal the interaction mechanism among the diverse complex components involved in the warm mix regeneration process. Molecular dynamics (MD) simulation provides an effective tool for exploring atomic interactions and thermodynamic behaviors during warm mix regeneration at the microscopic scale and facilitates the capture of intermolecular diffusion behaviors that are difficult to observe in conventional experiments [35].

**Figure 1:**
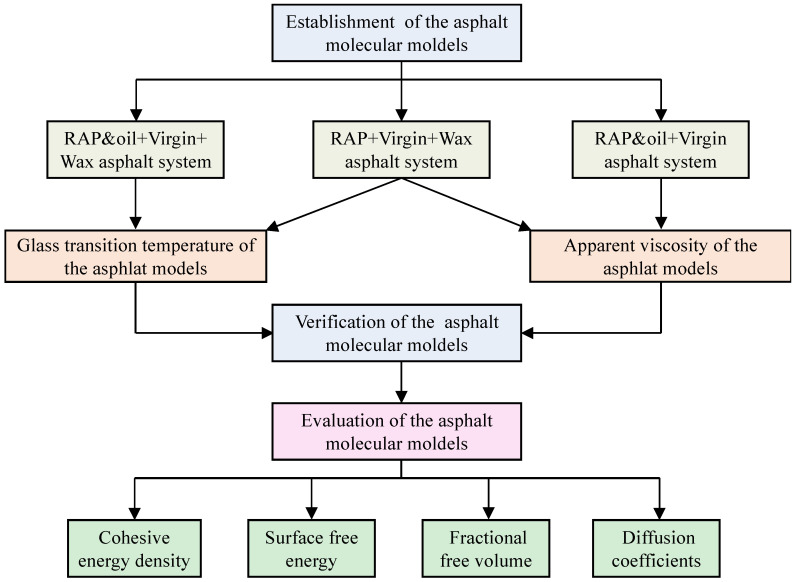
The research plan of this study.

For this purpose, a molecular dynamics model of warm mix recycled asphalt binder was designed to explore the effects of the dual action of wax-based additives and oil-based rejuvenators on the diffusion behavior and thermodynamic properties of virgin and RAP binders. The glass transition temperature and apparent viscosity simulated values of the warm mix recycled asphalt binder molecules were first compared with the experimental values to verify the correctness of the asphalt molecule modeling. Meanwhile, the cohesive energy density (CED), self-healing potential, fraction-free volume (FFV), and diffusion coefficient were computed to evaluate the difference between asphalt binders in thermodynamic properties and diffusion behavior after introducing the warm mix additives and rejuvenators. The findings of this paper aim to reveal the thermodynamic performance and microscopic diffusion mechanism of warm mix recycled asphalt with a high RAP binder content from the molecular level. The specific research plan for this research is presented in Figure 1.

## 2. Details of Molecular Dynamics (MD) Simulations

### 2.1. Establishment of the Asphalt Diffusion System

#### 2.1.1. Virgin Asphalt Binders

In this study, the Materials Studio (MS) 2019 version was used to develop a model of warm mix recycled asphalt binders with and without rejuvenators on a molecular scale. Asphalt binders are typically divided into four SARA components (saturate, asphaltene, resin, and aromatic) according to their physical characteristics and solubility behaviors [36]. Among the SARA components, asphaltenes are the heaviest and most complex components of asphalt binders, while the resin and aromatic fractions are generally considered unsaturated aromatics with strong and weak polarity, respectively. As for saturates, they consist of alkane chains or rings. The 12-component asphalt binder model is commonly chosen as the representative model for describing the virgin asphalt binder [37], including two components (squalane and hopane) for saturates, three components (asphaltene–phenol, asphaltene–pyrrole, and asphaltene–thiophene) for asphaltenes, five components (Quinolinohopane, Thioisorenieratane, Trimethylbenzeneoxane, pyridinohopane, and Benzobisbenzothiophene) for resins, and two components (PHPN and DOCHN) for aromatics [38]. Many of the proposed four-component molecular formulas have been demonstrated to exist in petroleum or nature. Hence, the 12-component asphalt binder model was applied for MS modeling in this study. Table 1 summarizes the detailed components and molecular formulas of the virgin asphalt binder. After establishing the 12-component asphalt binder model, we further specified the details of the virgin asphalt used in our simulations. This virgin asphalt is based on a common penetration grade of 70, which is also frequently used in typical asphalt mixtures.

#### 2.1.2. Aged Asphalt Binders

**Figure 2:**
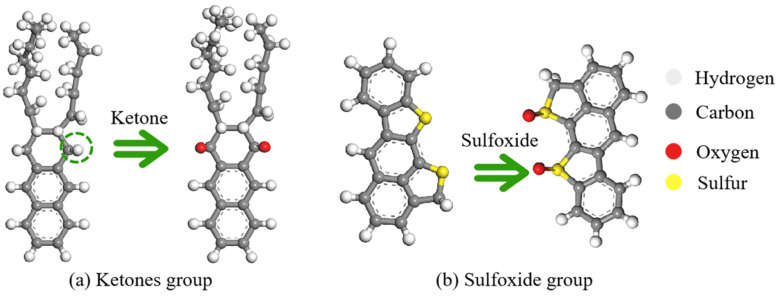
The typical oxidation products of asphalt binder after aging.

**Figure 3:**
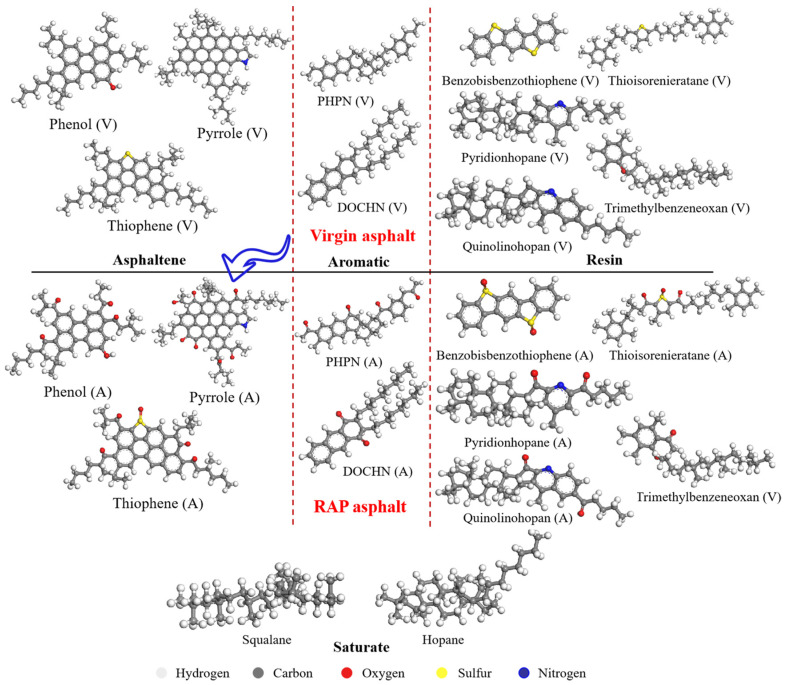
Molecular structures of 12-component asphalt binder model after long-term aging.

Asphalt pavements are continually subject to environmental influences, leading to irreversible alterations like chemical deterioration, the expulsion of volatile components, the depletion of lighter materials within aggregates, and stiffening as a result of temperature fluctuations, humidity, and traffic stress [39]. Among these, asphalt aging is mainly caused by oxidation, which happens in two stages [40]. At elevated temperatures, small alkanes rapidly volatilize, while alkyl groups on benzene rings undergo oxidation to form carboxyl groups, frequently converting long-chain alkyls into benzoic acid. As aging progresses, oxygen replaces hydrogen on benzyl carbon, forming ketones, which increases the concentration of ketones and sulfoxides. Figure 2 illustrates the major changes in the chemical structures of asphalt binders during the oxidative aging process. These changes result in a decrease in the content of resin and aromatic fractions, while asphaltenes increase sharply [41]. The complex aging processes drive the original stable colloidal structure of asphalt binders into an unstable one. Notably, the saturate fractions remain stable during oxidative aging, and this assumption has been widely used in previous molecular dynamics (MD) simulations [42]. Figure 3 shows the evolution of the asphalt molecules from the virgin binder to the RAP binder, and Table 1 records the components and molecular formulas of the RAP asphalt binders [43]. All aging processes were simulated at the molecular level, focusing on diffusion and thermodynamic behavior rather than fully capturing all physical processes.

**Table 1:** Molecular structures of the virgin and aged asphalt binders [43].

SARA	Model Numbers	Molecule Name	Virgin Asphalt		Aged Asphalt	
			Molecule Formula	Mass Fraction (%)	Molecule Formula	Mass Fraction (%)
Saturate	4	Hopane	C_35_H_62_	11.1	C_35_H_62_	10.3
	4	Squalane	C_30_H_62_		C_30_H_62_	
Aromatic	13	DOCHN	C_30_H_46_	31.9	C_30_H_42_O_2_	32.4
	11	PHPN	C_35_H_44_		C_35_H_36_O_4_	
Resin	4	Thioisorenieratane	C_40_H_60_S	39.8	C_40_H_56_O_3_S	39.6
	4	Pyridionhopane	C_36_H_57_N		C_36_H_53_NO_2_	
	4	Quinolinohopane	C_40_H_59_N		C_40_H_55_NO_2_	
	15	Benzobisbenzothiophene	C_18_H_10_S_2_		C_18_H_10_O_2_S_2_	
	5	Trimethylbenzeneoxane	C_29_H_50_O		C_29_H_48_O_2_	
Asphaltene	2	Pyrrole	C_66_H_81_N	17.3	C_66_H_67_NO_7_	17.7
	3	Phenol	C_42_H_54_O		C_42_H_46_O_5_	
	3	Thiophene	C_51_H_62_S		C_51_H_54_O_5_S	

#### 2.1.3. Warm Mix Asphalt (WMA) Additives

**Figure 4:**
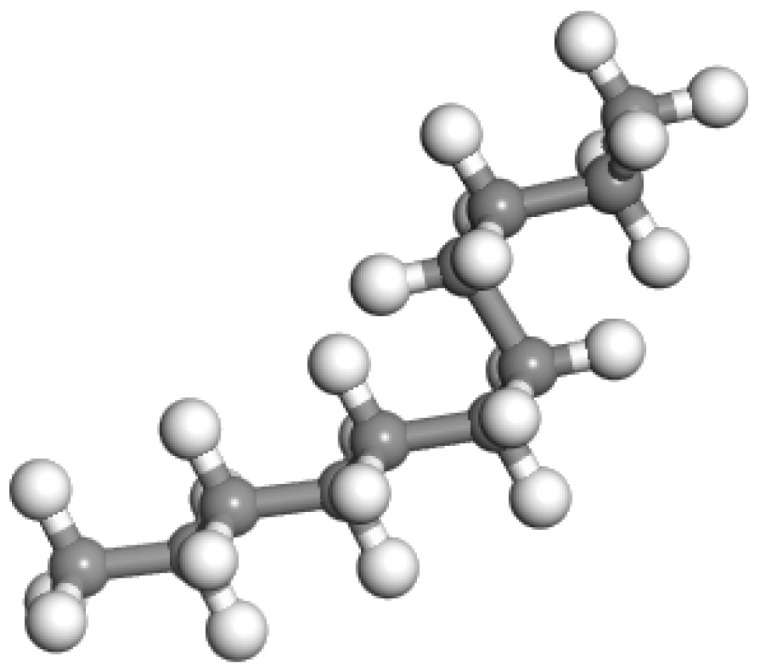
Molecular structures of wax WMA additive.

Organic wax is a commonly used WMA additive, as the presence of wax reduces the viscosity of bitumen, allowing it to liquefy at temperatures exceeding 100 °C. This results in a reduction in the mixing, incorporation, and compaction temperatures of the mineral and asphalt mixture, thereby achieving a satisfactory warm mix effect [44]. The basic building block of doped wax in this study was the n-paraffin wax model. This wax model, which has been utilized by other researchers [44], is typically comprised of n-C_11_H_24_. Figure 4 exhibits the molecular structures of the wax WMA additive. For this study, we selected the most commonly used warm mix asphalt (WMA) waxes, which have a micro crystallinity under 99~115 °C and a softening point of 99~115 °C.

#### 2.1.4. Rejuvenators

**Figure 5:**
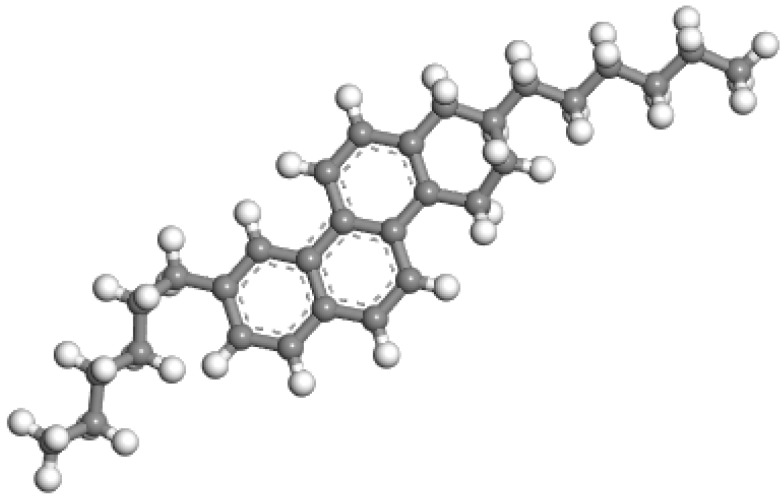
Molecular structures of aromatic oil rejuvenator.

In comparison to virgin asphalt binders, RAP asphalt binders generally have reduced levels of aromatic and saturated components. Thus, rejuvenators with simple structures composed of small molecules, either aromatic or saturate-based, are typically preferred. The aromatic oil rejuvenator (C_30_H_40_) is a commonly used rejuvenator, which is a complex mixture of several aromatic compounds, mainly from the refining of petroleum products. It is used for mixing into RAP asphalt binders, and its molecular configuration is illustrated in Figure 5 [45]. The basic properties of aromatic oil are exhibited in Table 2. The aromatic oil’s molecular model is straightforward, featuring just three benzene rings with methyl and methylene groups attached. To create an MD model for rejuvenated asphalt binders, aged and new asphalt binders were mixed in a box with 5% rejuvenators, a standard lab quantity [46]. The blend ratio of new to RAP (reclaimed asphalt pavement) asphalt binders was 1:1, forming a cube containing 72 new asphalt molecules, 72 RAP asphalt molecules, and 5 rejuvenator molecules.

#### 2.1.5. Mutual Diffusion System

**Figure 6:**
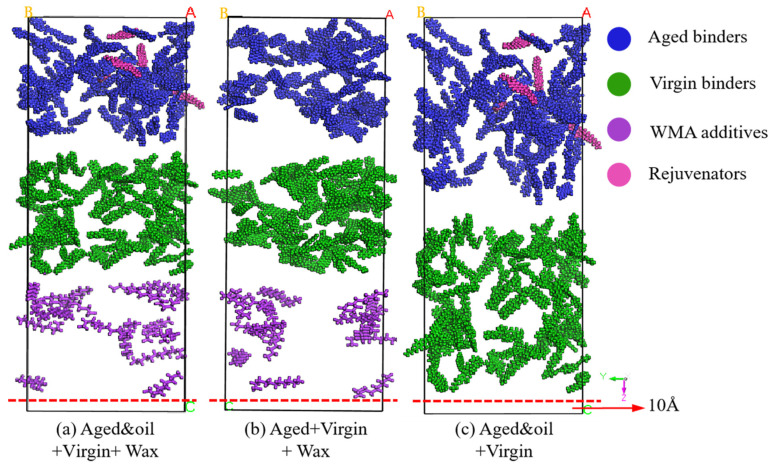
Three types of mutual diffusion systems.

To reveal the diffusion mechanism of warm mix recycled asphalt binders, three different mutual diffusion systems were established in MS (as shown in Figure 6). The Build Layers module in MS was used to assemble the RAP binders (blended with rejuvenators)–virgin binders–WMA additives as well as the RAP binders–virgin binders–WMA additives three-layered mutual diffusion system, respectively. In addition to this, a double-layered mutual diffusion system of RAP binders (blended with rejuvenators)–virgin binders was developed as the control group to study the effects of the WMA additive on the interdiffusion between aged and virgin asphalt molecules. In the system containing rejuvenators and WMA additives, the aged binders layer consisted of 72 RAP asphalt molecules and 5 rejuvenator molecules, and the virgin binders included 72 unaged asphalt molecules, while the third layer comprised 14 wax WMA molecules (equal to 3% of the total binder’s content). As depicted in Figure 6, a vacuum layer of 10 Å was added on the Z-direction edge side of the diffusion, indicating that the Z direction was assigned a non-periodic condition, while the X and Y directions retained their periodic boundaries.

### 2.2. MD Simulation Procedures

**Figure 7:**
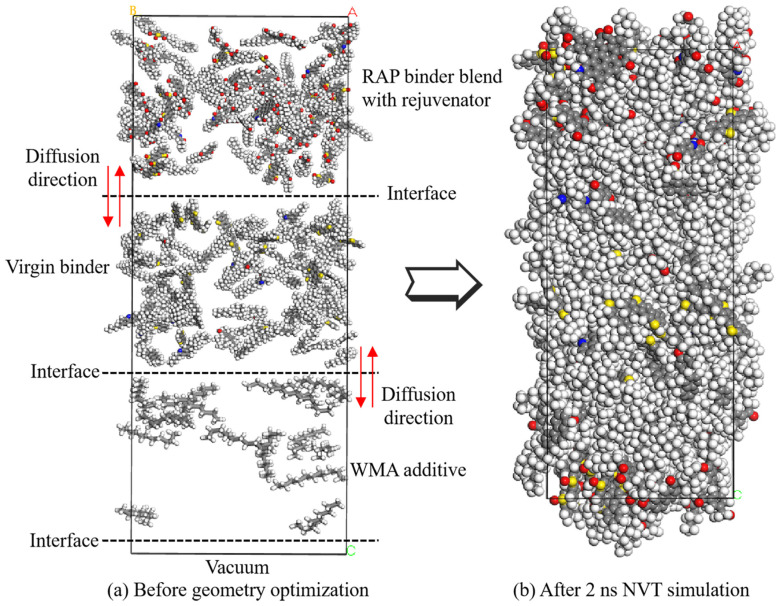
Illustration of warm mix recycled asphalt molecule system (Aged and oil + virgin + wax as an example).

To prevent overlapping and twisting, all molecules were first dispersed in a rectangular box with an initial density of 0.1 g/cm^3^, including the RAP binders with and without rejuvenators, virgin binders, and WMA additives. The Build Layers module was utilized to construct the mutual diffusion systems. Subsequently, the geometry was optimized using the smart descent algorithm over 5000 iterations. Proceeding to the simulations, a 500 ps MD simulation was conducted under NVT conditions to attain a target temperature of 433.15 K. This was followed by another 500 ps MD simulation under NPT conditions. Finally, the thermal properties were calculated and evaluated using a two-nanosecond NVT simulation. The NHL thermostat was used to control temperature, and the pressure was set at 1.0 atm (0.000101325 Gpa) using the Andersen barostat. Figure 7 shows the depiction of the warm mix recycled asphalt molecule system at both the beginning and the end of the simulation.

## 3. Evaluation Indexes

### 3.1. Glass Transition Temperature

Asphalt behaves in a glassy state at low temperatures and gradually transforms into a rubbery state as the temperature increases [47]. The glass transition temperature (T_g_) is the critical temperature at which the asphalt binder changes from the glass state to the rubber state, which is an essential index to predict the low-temperature behavior of asphalt and its mixtures. In this paper, the T_g_ of the three asphalt systems was tested and simulated. The T_g_ kinetic simulation procedure consists of the following: Initially, the three asphalt systems underwent a 500 ps NPT MD simulation from 183.15 K to 393.15 K at intervals of 30 K. The specific volumes of the three asphalt systems were then calculated at different temperatures based on the asphalt binder density obtained from the simulation results. Finally, the T_g_ of each asphalt system was determined by analyzing the specific volume–temperature curve. Meanwhile, the simulated T_g_ values were verified by performing Differential Scanning Calorimetry (DSC) (TA Instruments, USA) tests. During DSC testing, the testing temperature ranged from −80 °C to 80 °C, with a heating rate of 10 °C/min. Apparently, the low T_g_ temperature corresponds to the better low-temperature cracking resistance of asphalt and its mixtures [47].

### 3.2. Asphalt Binder Viscosity

Viscosity is a fundamental viscoelastic property of asphalt binder that is closely related to the mechanical performance of asphalt pavement. In this study, the asphalt viscosity was calculated by employing the Williams–Landel–Ferry (WLF) model based on the simulated glass transition temperature [48]. The detailed expression of the asphalt model viscosity computation is exhibited in Equation (1):

(1) $$\mathrm{lg}\frac{\eta (T)}{\eta (T_{g})}=-\frac{17.44(T-T_{g})}{51.6+(T-T_{g})}$$

where η(T) is the asphalt viscosity at the temperature of T (K); and $\eta (T_{g})$ is the asphalt viscosity at the glass transition temperature (K) at about 10^12^ Pa·s.

Meanwhile, the actual asphalt viscosity of the three asphalt binders was captured by using Brookfield Rotational Viscometer tests (Shanghai Changji Geological Instrument Co., Ltd., Shanghai, China) (NDJ-79) outlined in AASHTO T316. The objective was to verify the reasonability of the MD simulated viscosity values.

### 3.3. Cohesive Energy Density (CED) and Surface Free Energy (SFE)

Cohesive energy density (*CED*) is the average energy required to overcome intermolecular forces when 1 mol of condensed material is vaporized per unit volume [48]. It is commonly used to evaluate intermolecular interactions in asphalt models. *CED* is usually derived using Equations (2) and (3) [49]:

(2) $$CED=\delta^{2}$$

(3) $$\delta =\sqrt{\delta_{vdw}^{2}+\delta_{ele}^{2}}$$

where δ represents the solubility parameter; and $\delta_{vdw}$ and $\delta_{ele}$ designate the electrostatic solubility and van der Waals solubility of asphalt binders, respectively.

In general, a higher number of macromolecules in an asphalt model indicates a stronger intermolecular interaction force, leading to a larger *CED* [48]. Meanwhile, the smaller solubility parameters indicate better compatibility for the asphalt molecular model. Similar to *CED*, the workforce of separation that produces a new surface per unit of solid or liquid, called surface free energy (*SFE*), is widely employed to estimate the cracking resistance of an asphalt binder. Evidently, a higher *SFE* corresponds to satisfactory cracking resistance [49]. The definition of *SFE* is exhibited in Equation (4):

(4) $$\gamma_{a}=\left(E_{film}-E_{bulk}\right)/2A$$

where $\gamma_{a}$ represents the *SFE*; $E_{film}$ stands for the potential energy of the confined asphalt binder; $E_{bulk}$ is the potential energy of the bulk asphalt binder; and A corresponds to the percentage of asphalt binder in X and Y directions.

### 3.4. Asphalt Binder Density

Due to the influence of complex environmental factors and heavy traffic loads, an asphalt binder is susceptible to the risk of cracking during its service life [50]. These microcracks in asphalt binder can be repaired at a certain temperature threshold, which is called the self-healing process of asphalt binder [50]. In the asphalt molecular system, this self-healing process is generally characterized by changes in the asphalt density [48]. As exhibited in Figure 7, the 10 Å vacuum middle layers set in the asphalt molecular system were used to simulate microcracks in the asphalt binder. To capture the self-healing process, an MD simulation of 500 ps was conducted by employing the NPT ensemble at 1 atm and 298 K. During the simulation, the mutual diffusion between the upper and bottom layers of the asphalt molecular system was used to stimulate self-healing. When the density of the asphalt molecular system reached a stable level, it was considered fully self-healed. Thus, the time taken to reach a stable level was used as an evaluation index to assess the self-healing ability of warm mix recycled asphalt binders with and without rejuvenators.

### 3.5. Fractional Free Volume

The mobility and permeability of asphalt materials at the atomic level are often assessed quantitatively by fractional free volume (*FFV*) [51]. The presence of *FFV* enables molecular chains to undergo rotations and displacements [51]. The definition of *FFV* is defined as exhibited in Equation (5):

(5) $$FFV=\frac{V_{free}}{V}=\frac{V_{free}}{V_{free}+V_{occupied}}$$

where *FFV* is the fractional free volume; *V* is the molecule volume; *V_free_* stands for the free molecule volume; and *V_occupied_* stands for the occupied molecule volume.

**Figure 8:**
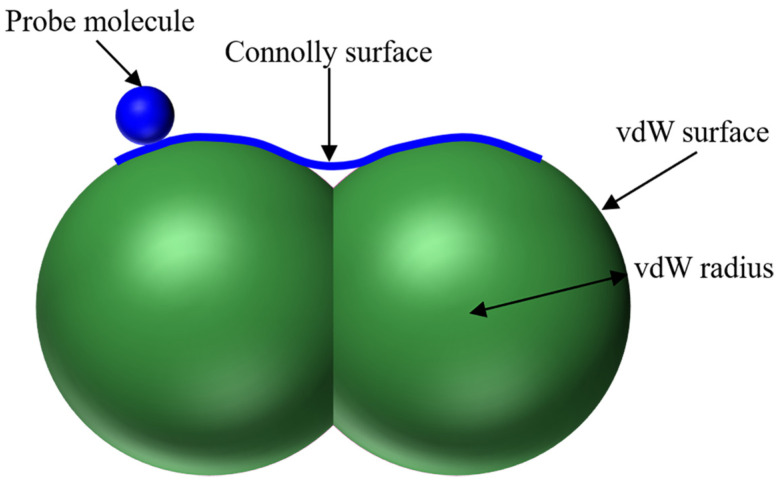
Schematic diagram of the Connolly surface method for determining *FFV*.

The Connolly surface method is a typical measurement approach to determine the *FFV* value [49]. The measurement principle is summarized as follows:

The Connolly surface, as depicted in Figure 8, is generated by moving probe atoms of radius R_P_ along the exterior of these atoms within the asphalt molecular system. Based on this, Equation (5) is further transformed into Equation (6):

(6) $$FFV=\frac{V-V_{occupied}}{V}=\frac{V-1.3V_{vdW}}{V}$$

where *V_vdW_* represents the van der Waals volume.

The *FFV* exerts a decisive influence on the diffusion and glass transition behavior of molecules. Thus, the *FFV* of the three developed asphalt molecule models was calculated to evaluate the rheological properties of the warm mix recycled asphalt binders with and without rejuvenators.

### 3.6. Diffusion Coefficients

During the MD simulations, the asphalt molecules of the four SARA components move continuously from their initial position until a dynamic equilibrium is achieved. The warm mix regeneration process between the RAP binders and the virgin binders was highly influenced by the interaction of the WMA additives and rejuvenating agents. Hence, it is crucial to explore the diffusion characteristics of the SARA components. To determine the distributions of the SARA components at the molecular level, the mean square displacement (*MSD*) and self-diffusion coefficient (*D*) were calculated using Equation (7):

(7) $$D=\frac{1}{6N}\underset{t\to \infty }{\lim}\frac{d}{dt}\sum_{i=1}^{N}MSD(t)=\frac{1}{6N}\underset{t\to \infty }{\lim}\frac{d}{dt}\sum_{i=1}^{N}\langle {\left|r_{i}(t)-r_{i}(0)\right|}^{2}\rangle$$

where *N* is the number of asphalt atoms; *r_i_*(*t*) designates the location of atom *i* at the time of *t*; *r_i_* (0) accounts for the initial location of atom *i*; and the angle brackets are a flag to calculate the ensemble average of the *MSD*.

As for the mutual diffusion system, the asphalt binder, organic wax, and rejuvenator molecules tend to spread and intermix themselves until they achieve a homogeneous distribution. The density curve serves as a numerical representation of the spatial arrangement and concentration of a particular molecule. Hence, in this part, the Z directional density curves of the designed three asphalt molecule systems were detected during the MD simulations to estimate the mutual diffusion coefficient (*D*_0_), as expressed in Equation (8):

(8) $$\rho (z,t)=\rho_{0}(1-erf(\frac{z}{2\sqrt{D_{0}t}}))$$

where ρ(z,t) means the density curves; $\rho_{0}$ represents the equilibrium mass density of waxes or rejuvenators; *erf* refers to the error function; *z* denotes the location of waxes or rejuvenators in the Z axis (i.e., molecule diffusion direction); and *t* represents the simulation time.

## 4. Results and Discussion

### 4.1. Glass State Transition Behavior

**Figure 9:**
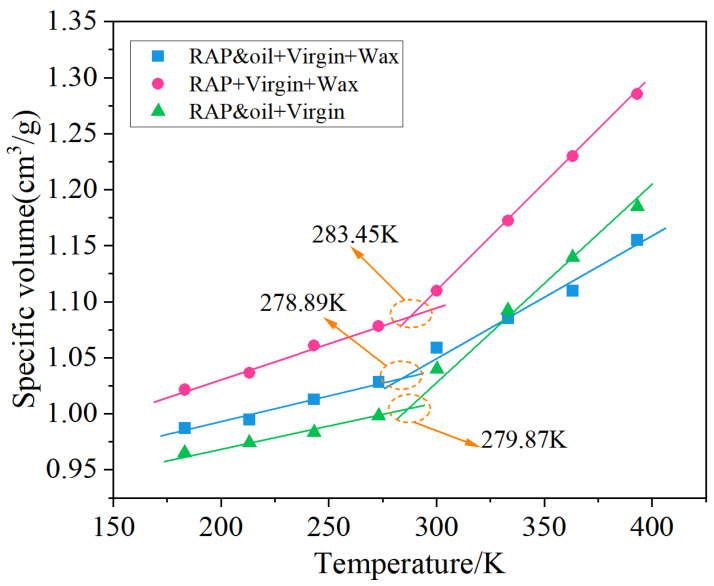
Determination of glass transition temperatures from MD simulations.

The glass transition temperature (T_g_) of an asphalt binder is a significant index in evaluating the complex low-temperature behavior of asphalt and its mixtures [52]. In this section, the specific volumes of each asphalt system were computed based on the corresponding simulated asphalt density results (the reciprocal of density). The data points for volume–temperature relationships in various zones were individually modeled, as illustrated in Figure 9. T_g_ points are determined by analyzing the characteristics of these fitting curves, marking them as the transition between the glassy and viscoelastic regions [48]. Generally, the asphalt binder with a low T_g_ corresponds to better cracking resistance at low temperatures [52].

As illustrated in Figure 9, the simulated T_g_ values of RAP and oil + virgin + wax asphalt system, RAP + virgin + wax asphalt system, and RAP and oil + virgin asphalt system are 278.89 K (5.74 °C), 283.45 K (10.3 °C), and 279.87 K (6.72 °C), respectively, all falling in the normal glass transition temperature range (i.e., 223–303 K) reported in the previous literature [49]. The presence of organic wax WMA additives and RAP binders increases the T_g_ value, while the introduction of oil rejuvenators offsets this effect. The T_g_ of the bitumen used in this study was measured under specific conditions and may vary depending on the composition and additives employed. While a T_g_ greater than 0 °C may seem high, it is important to note that the bitumen selected for this study was chosen for its particular characteristics relevant to warm mix asphalt (WMA) performance, and it may still provide effective mixing and compaction temperatures under the conditions tested. This highlights the importance of using oil rejuvenators along with WMA additives to achieve desired low-temperature cracking resistance in warm mix recycled asphalt binders. It can be concluded that the presence of organic wax WMA additives and RAP binders increases the value of T_g_, and the introduction of oil rejuvenators offsets the detrimental effects on T_g_ value. The increase in T_g_ indicates the glass transition of asphalt and its mixtures will undergo at higher temperatures, leading to the earlier cracking of asphalt pavements. For the RAP + virgin + wax asphalt system with the highest T_g_ value, it may be ascribed to the presence of oxidatively aged RAP binders, which contributed to the increase in stiffness to the asphalt system, inducing the glass transition at a not-very-low temperature. As reported in previous studies, the oxidative aged RAP binders raise the T_g_ from 271.96 K to 292.73 K [49], demonstrating that the risk of asphalt cracking increased a lot. Additionally, organic waxes behave as solids at room or lower temperatures, which also favors the increase in asphalt stiffness. Consequently, the dual contributions of organic waxes and aged RAP binders to asphalt stiffness result in the highest T_g_ value of the RAP + virgin + wax asphalt system. After the rejuvenating agent was incorporated into the asphalt system, the T_g_ value decreased from 10.3 °C to 5.74 °C. This indicates that the addition of rejuvenating agents significantly improves the anti-cracking resistance. This is mainly attributed to the presence of aromatic oil softening the stiff organic waxes and aged RAP binders, and, thus, the glass transition temperature of the RAP and oil + virgin + wax asphalt system decreased largely. In conclusion, oil rejuvenators must be added alongside WMA additives in warm mix recycled asphalt binders to enhance resistance to low-temperature cracking.

**Figure 10:**
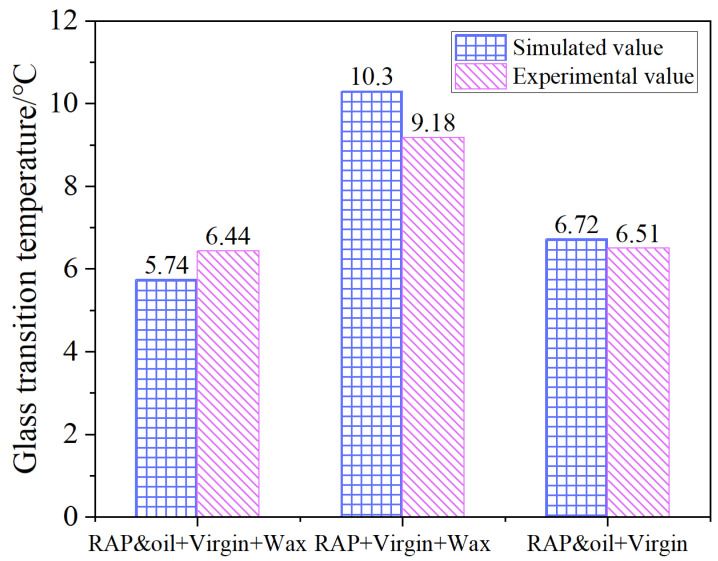
Glass transition temperature comparisons between DSC testing and MD simulation results.

The comparison of simulated and experimental T_g_ values of the three asphalt systems is presented in Figure 10, and the relative errors are within 12.2%. Hence, it can be concluded that the simulated and experimental values have satisfactory consistency, demonstrating the MD simulations in predicting glass state transition behavior are feasible, and the MS model developed in this study is reasonable as well.

### 4.2. Viscosity Simulation Prediction

**Figure 11:**
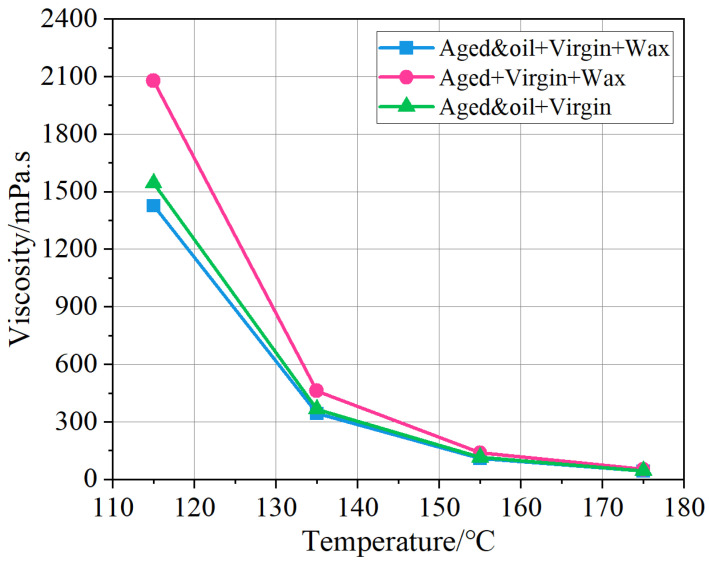
The viscosity of different asphalt systems calculated by the MD simulation results.

Through the WLF model described in Section 3.1, the apparent viscosities of the warm mix recycled asphalt binders with and without rejuvenators were calculated, and the viscosity change curves at different temperatures were plotted, as exhibited in Figure 11. As can be discovered in Figure 11, all the viscosity decreases with increases in temperature because the forces between asphalt molecules gradually weaken at high temperatures. At the same temperatures, the viscosity of the RAP + virgin + wax asphalt system is the highest among the three asphalt binders, and the other two asphalt binders show a relatively low viscosity. This phenomenon reveals that the asphalt binder with wax but without rejuvenators cannot become a WMA binder with low viscosity. In other words, the presence of organic wax does not appear to play a role as a WMA additive if rejuvenators are not introduced into the asphalt binder. It is due to the fact that the RAP binders, without any pretreatment, bring more macromolecular such as asphaltene and resin into the asphalt system. After oxidative aging, some saturate and aromatic in the asphalt system were transferred into asphaltene and resin, leading to an increase of heavy components in RAP binders. These heavy components provide more viscosity to the asphalt molecules and thereby weaken the warm mix performance of organic waxes. After the addition of rejuvenators, the viscosity of the RAP and oil + virgin + wax asphalt binder is conspicuously decreased, indicating a better workability performance at the same temperature compared to the RAP + virgin + wax asphalt binder. Evidently, this is attributed to the fact that the incorporation of aromatic oil replenishes some light components in the asphalt system and avoids the detrimental influence of aged heavy components on asphalt viscosity. Meanwhile, organic wax molecules are completely soluble in light asphalt molecules at the temperature of 115 °C, adsorbing the saturate components of the asphalt binder that are similar in structure to it (a portion of the wax- or oil-based molecule) and dissolving in the saturate component, thus forming a stable solution. Under this adsorption and dissolution, the asphalt binder accelerated melting, and the higher the temperature, the more pronounced the adsorption, leading to a reduction in the high-temperature viscosity of the asphalt binder. To determine the threshold values, a quadratic regression model using the least squares method was applied to fit the viscosity data. As presented in Figure 11, the RAP and oil + virgin + wax asphalt binder manifests the lowest viscosity among these asphalt binders.

In addition, Table 3 lists the comparisons of the simulated and experimental viscosity at different temperatures. It can be seen that the relative error between the simulated and experimental viscosity is within 11.2%, suggesting the MD simulations in predicting asphalt viscosity is feasible, and the MS model developed in this study is also reasonable.

### 4.3. Thermal Properties

The thermal properties of the asphalt molecular systems at the simulation temperature of 433.15 K are presented in Table 4. Density stands as a key thermodynamic property of asphalt binders among these attributes. The simulated density results point out that the density of the Aged + virgin + wax asphalt system is a little higher than the other two systems, ascribed to the aged RAP binders without rejuvenation. Oxidative aging of the asphalt binder means the introduction of oxygen atoms, as described in Figure 2, which is beneficial to the increase in density. Thus, compared to the Aged and oil + virgin + wax asphalt system, the density of the asphalt system without rejuvenating oil is increased by 2.1%. As for the density of the Aged and oil + virgin asphalt binder, it is almost consistent with the Aged and oil + virgin asphalt binder, implying the addition of organic wax to the density is not significant. Meanwhile, these discussed density values basically remain consistent with the density values reported in previous studies, which also verifies the effectiveness of the MD simulations in this study.

Other thermodynamic indices are summarized in Table 4. CED serves as the evaluation metric for assessing the intermolecular forces within the asphalt molecular structure. Evidently, the CED value for the Aged + virgin + wax asphalt system is 3.162 × 10^8^ J/m^3^, which is increased by 2.86% in comparison to the asphalt system with rejuvenation by aromatic oil. In general, asphalt binders with large CED values diffuse slower under the same conditions, which is macroscopically manifested as a rise in asphalt viscosity. This is because there is an increase in the proportion of macromolecular asphaltene and resin in RAP binders. Similarly, the solubility parameters (δ, δ_vdw_, and δ_ele_) of the Aged + virgin + wax asphalt system also increase, which is caused by the enhanced electrostatic interaction of asphalt molecules. After blending with small molecules of aromatic oil, the CED values and solubility parameters all reduced, suggesting dissolution of the aromatic rejuvenators in the RAP binder reduces the macromolecular interaction force. Hence, the diffusion performance of the asphalt binder is expected to be improved to some extent, and this phenomenon will be discussed in the following sections.

The SFE represents the energy required to separate the block-shaped asphalt and regenerate a new surface, indicating the strength of intermolecular cohesive forces in asphalt molecules. As illustrated in Table 4, the surface energy of the Aged + virgin + wax asphalt binder is lower than the WMA binder rejuvenated by aromatic oils, which means the required energy for producing a new surface is less, and cohesive work is also low without the presence of rejuvenators. It may result in the Aged + virgin + wax asphalt binder being susceptible to cohesive failure and cracking in the same conditions. The incorporation of 5% rejuvenators no doubt increases the surface energy to a large extent, with the SFE rising from 18.5 mJ/m^2^ to 36.4 mJ/m^2^. This demonstrates that aromatic oils boost the adhesive strength of the RAP binder, thereby improving durability against cracking in the asphalt binder. The SFE of the Aged and oil + virgin + wax asphalt binder is basically consistent with the aromatic oil-rejuvenated asphalt binder, which is slightly lower than the asphalt binder without WMA additives. This may be due to the organic wax decreasing the cohesive work, but this detrimental influence is slight.

### 4.4. Self-Healing Ability

**Figure 12:**
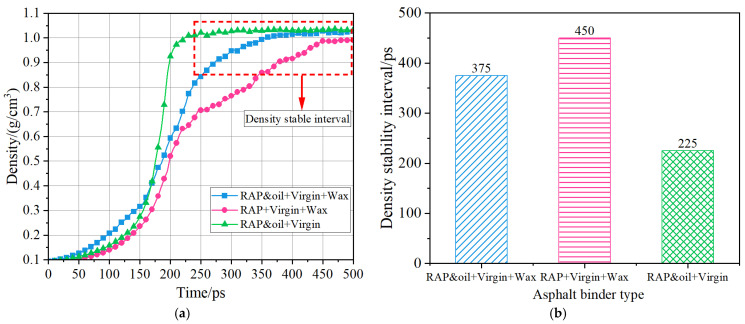
Density curves of the three asphalt binders after the 500 ps MD simulations (298.15 K): (**a**) density curves; (**b**) density stability interval.

As detailed in Section 3.4, identical microcracks were created in all three asphalt molecular systems, and the NPT molecular dynamics simulations were subsequently performed on these three systems. The density curves of the three systems during the 500 ps simulations are drawn in Figure 12a. As the density curve of the asphalt binder stabilizes, it can be regarded as indicating fully self-healed microcracks. Evidently, different asphalt molecular systems have different self-healing abilities, as presented in Figure 12b. With regard to the Aged + virgin + wax asphalt binder, the self-healing time is about 450 ps, which is the longest one among the three asphalt binders. This indicates that the self-healing performance of the Aged + virgin + wax asphalt binder is poorer than the other two asphalt binders. After oxidative aging, the small and medium molecules in the RAP binders (i.e., aromatic molecules and saturate molecules) were gradually converted into macromolecules (asphaltenes and resins). Due to this change, the reduction in light components in the Aged + virgin + wax asphalt binder causes a decrease in asphalt fluidity, thereby leading to a low self-healing rate. As presented in Figure 12, it is evident that incorporating aromatic oils into the Aged + virgin + wax asphalt binder enhances its self-healing capabilities, as indicated by the earlier attainment of density stabilization (375 ps). This accounts for the presence of aromatic oils replenishing some small aromatic molecules into the asphalt binder, thus improving the asphalt fluidity. Note that the self-healing time of the Aged and oil + virgin asphalt binder is the shortest at 225 ps. This may be due to the fact that the organic waxes presented as solids at the simulated temperature of 298.15 K increased the stiffness of the asphalt, which is not favorable for the flow of the asphalt binder. Hence, the asphalt binder, without the incorporation of organic waxes, manifests the best self-healing performance. It is important to mention that the density values presented in Figure 12 do not align with the density change trend observed in Table 4, potentially resulting from the disparity in simulated temperatures.

### 4.5. Changes in Free Volume

**Figure 13:**
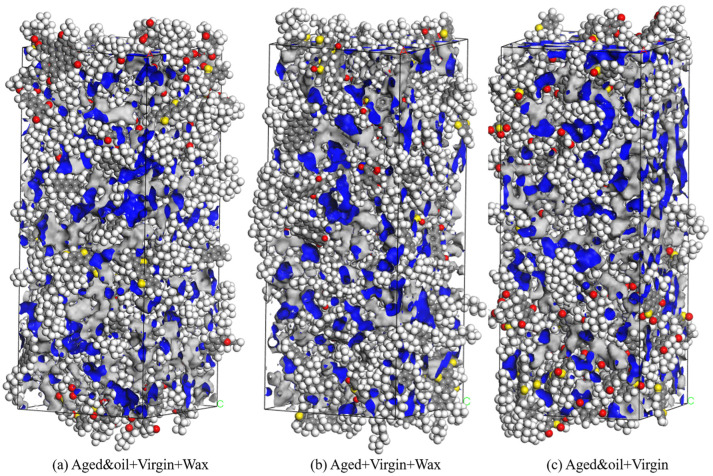
Visualization of the free volume of the three asphalt binders (free volume: blue parts; occupied volume: grey parts).

The investigation of the free volume offers valuable insights into the movement and distribution of the four SARA components within asphalt. Figure 13 clearly illustrates the spatial arrangement of the free volume (represented by blue areas) within the three asphalt molecular systems. It is evident that the free volume is nearly evenly spread across the gaps in the asphalt molecular system. Table 5 describes the calculated values of the occupied volume (grey parts), free volume, and FFVs of the three asphalt molecular systems. The free volume of the asphalt binder with organic waxes but without rejuvenators slightly decreased (from 22.02% to 21.9%) in comparison to the asphalt binder with organic waxes and rejuvenators, implying that the asphalt molecular structure becomes more compact, increasing the van der Waals volume and reducing the gaps between unoccupied molecules. This phenomenon is plausible, and it has also been reported in many previous studies [49]. When an appropriate number of rejuvenating agents is added, the dosage of nonpolar or low-polar aromatics in the RAP binder is enriched again, positively influencing the increase in free volume, thereby enhancing the rheological properties of the asphalt binder. Furthermore, the asphalt binder with rejuvenators but without WMA additives manifests the lowest free volume (21.03%) among these three binders. A possible explanation is that organic waxes adsorb and dissolve in the wax- or oil-based molecules in the asphalt binder at high temperatures (433.15 K equals 160 °C), thus accelerating the melting and movement of the asphalt molecules, which helps increase the free volume of the asphalt binder. Consequently, this phenomenon leads to the worst free volume of the asphalt binder with rejuvenators but without WMA additives.

### 4.6. Diffusion of Four SARA Components

**Figure 14:**
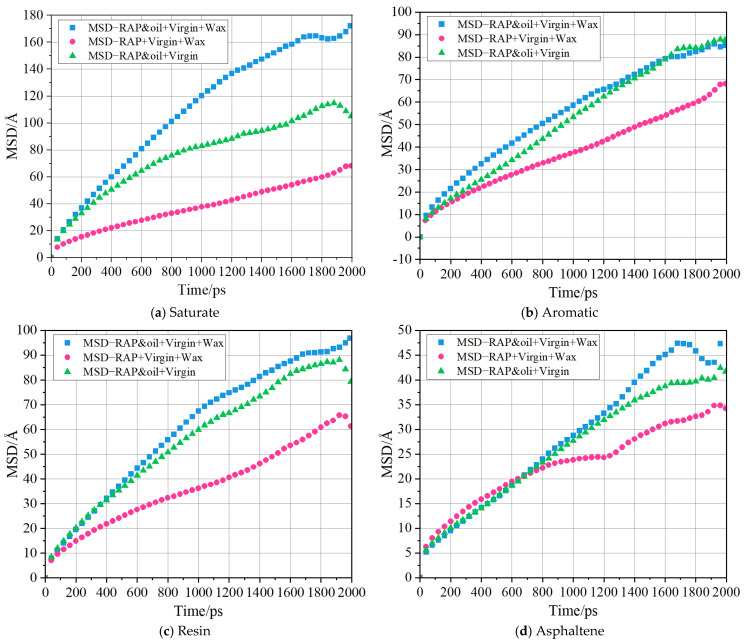
MSD curves of the SARA components at the end of MD simulations.

Given that self-diffusion is measured with the four SARA components of asphalt randomly arranged in a specified space, studying their diffusion behavior can provide insight into the mechanisms by which organic waxes and rejuvenating agents interact with and affect the performance of warm mix recycled asphalt binders. By plotting the self-diffusion curves of the SARA components during MD simulations, it is possible to distinguish the intrinsic characteristics of different types of asphalt binders. The MSD curves of the SARA components at the simulation temperature of 433.15 K are drawn in Figure 14. From Figure 14, it can be seen that the self-diffusion curves of the three binders are approximately straight and rise with increasing simulation times. And the location of the MSD curves varies for different asphalt binders. The MSD curves of the asphalt binder with organic waxes but without rejuvenators are below the other two binders. Although the final stage of the simulation shows small fluctuations, the linear fit results of the MSD curves in the Cartesian coordinate system are still acceptable, which is attributed to the long simulation time span (2 ns). Hence, the entire 2 ns simulation period was selected for calculating the MSD slope. Subsequently, the self-diffusion coefficients of each component were calculated by referring to Equation (7), as exhibited in Figure 14.

**Figure 15:**
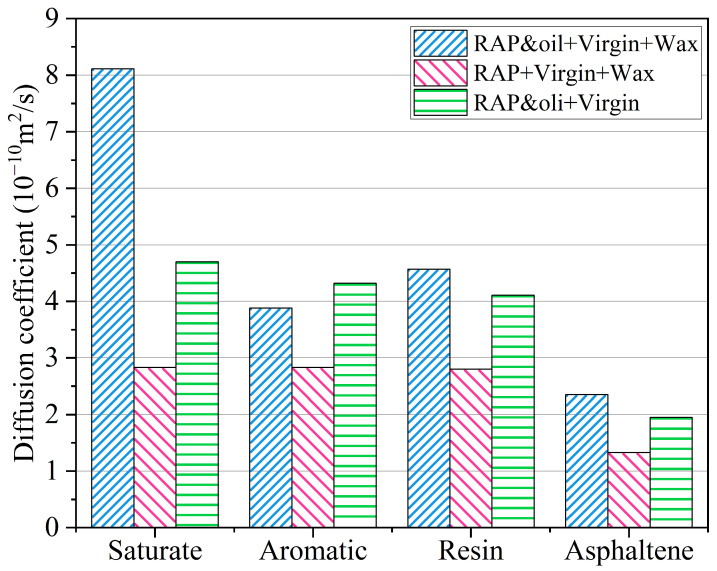
Self-diffusion coefficients of SARA components in the three asphalt binders.

As illustrated in Figure 15, each SARA component in the asphalt binder undergoes diffusion in an approximation based on its molecular weight due to a combination of intermolecular forces and thermodynamic motion. Owing to their small molecular weight and simple molecular structure, saturated substances have the highest self-diffusion rates, followed by resins and aromatics. Asphaltene manifests the slowest self-diffusion velocity as a result of its significant molecular mass, intricate structure, and tendency to self-assemble driven by π-π stacking interactions involving benzene rings. Furthermore, the self-diffusion coefficients of the asphalt binder with organic waxes but without rejuvenators are the lowest among these three asphalt binders. It accounts for the presence of aged RAP binder in the RAP + virgin + wax asphalt binder. After the oxidative aging of asphalt molecules, the volume and content of the resin and asphaltene increase. It leads to the FFV of aged asphalt becoming smaller, i.e., the unoccupied volume becomes smaller (as discussed in Section 4.5), implying that the aged RAP binder lacks sufficient room for the diffusion of SARA molecules. After incorporating 5% aromatic oils, the observations show a significant improvement in the self-diffusion coefficients of the RAP and oil + virgin + wax asphalt binder. It illustrates that the rejuvenators may provide an effective space for the diffusion of the SARA components and, meanwhile, improve the compatibility of WMA additives with asphalt molecules, substantially facilitating the degree of self-diffusion of the SARA components.

**Figure 16:**
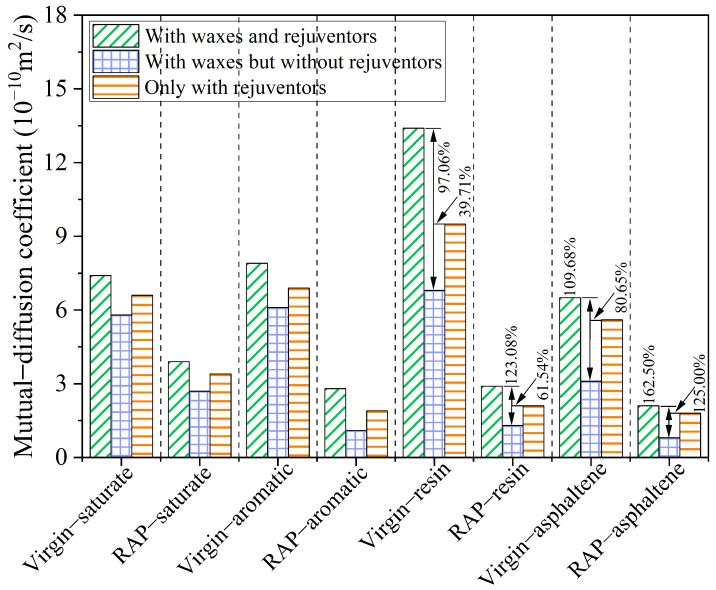
Mutual diffusion coefficients of SARA components in the three asphalt binders.

The blending process between the virgin and RAP asphalt binder is referred to as mutual diffusion, ascribed to the interaction of the different types of molecules in the asphalt system. Exploring the mutual diffusion of the asphalt molecular system is conducive to revealing the regulation behavior of WMA additives and rejuvenating agents in the blending process of virgin and RAP asphalt binders. Hence, in addition to the self-diffusion coefficients, the mutual diffusion coefficients of the SARA components are also calculated using Equation (8), as demonstrated in Figure 16. As depicted in this figure, the diffusion coefficient of the virgin binders is generally higher than that of the RAP binders, indicating that the blending of the two binders occurs primarily through the diffusion of the virgin binders. The SARA molecules in the mutual diffusion system manifest a marginally different diffusion behavior in comparison to self-diffusion. The resin evolves into the most active fraction, and its diffusion velocity exceeds that of light components such as aromatic and saturate fractions. Additionally, asphaltenes still maintain the slowest diffusion velocity of the three asphalt molecular systems, especially for the asphalt molecular system containing organic waxes but no rejuvenators. The mutual diffusion coefficient of virgin asphaltene in the Aged + virgin + wax asphalt binder is 3.1 × 10^−10^ m^2^/s, while the aged asphaltene is only 0.8 × 10^−10^ m^2^/s. As discovered in 4.5, without the presence of rejuvenators, the high polar oxygen atoms that have undergone oxidative aging generate stronger intermolecular forces, resulting in the self-aggregation of the RAP asphaltenes. An increase in the viscosity of RAP binders significantly influences the diffusion and flow behavior of long-chain molecules like resins and asphaltenes. Under these conditions, the mutual diffusion between the RAP and virgin binders is notably slow, particularly for the RAP components. This fact suggests that the de-aggregation of aged asphaltenes may be the key to improving the full blending of virgin and RAP asphalt binders.

**Figure 17:**
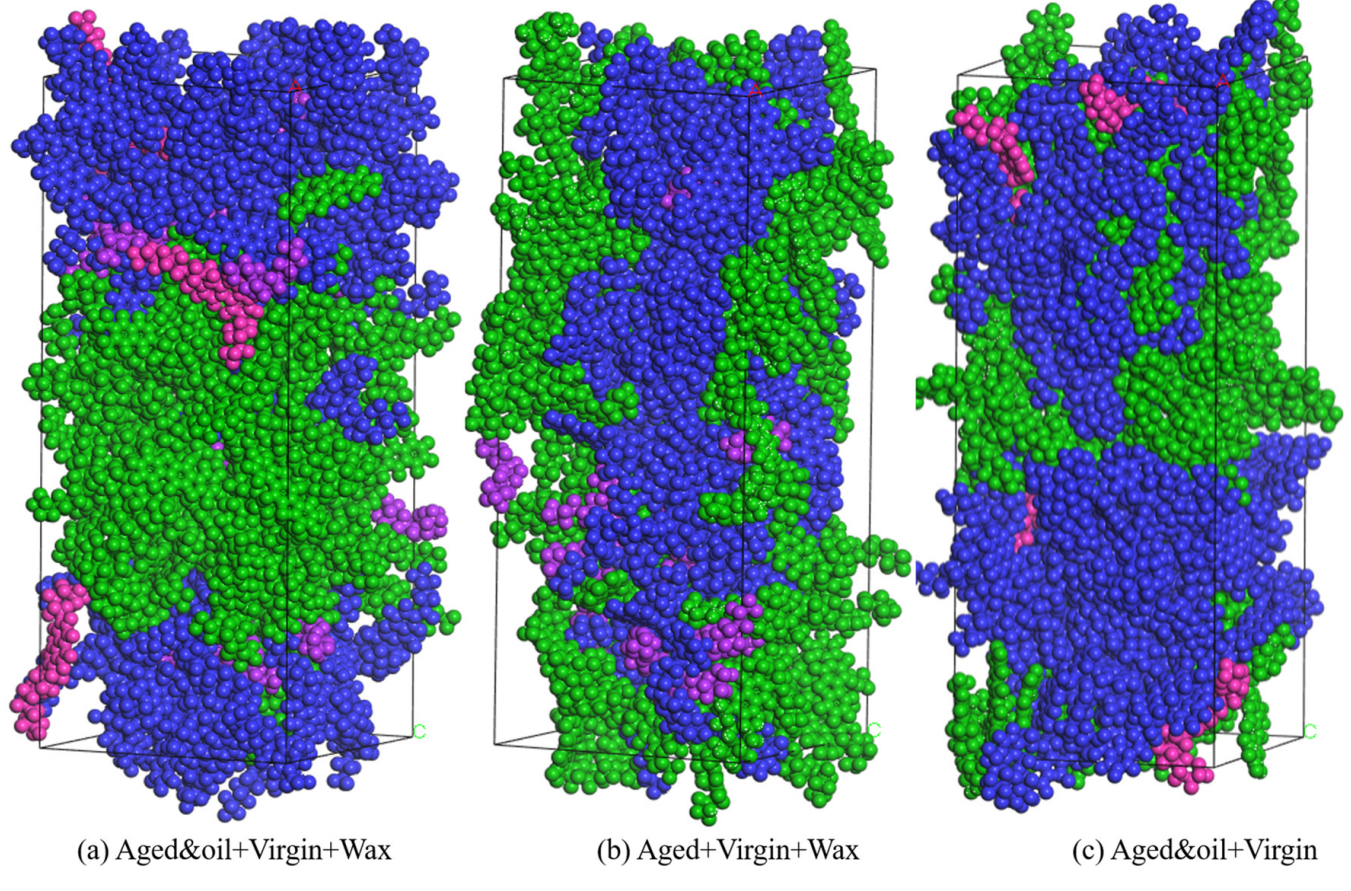
Mutual diffusion systems of the three asphalt binders at the end of MD simulations.

After adding 5% aromatic oil to the diffusion system, the blending efficiency of the virgin and RAP binders significantly improves, and the diffusion coefficient of the SARA molecules is remarkably enhanced as well, especially for the resin and asphaltene, in which, the virgin and RAP resin molecules in the Aged and oil + virgin + wax asphalt binder are increased by 97.06% and 123.08%, respectively. Meanwhile, the virgin and RAP asphaltene molecules are amazingly raised by 109.68% and 162.50%, respectively. At this time, the mutual diffusion coefficients of the Aged and oil + virgin + wax asphalt binder are the largest among these three asphalt systems. It shows that the concurrent presence of organic waxes and rejuvenators stimulates the activity of SARA components, particularly the aged SARA macromolecular components. Obviously, the activity of macromolecules plays a decisive role in the whole mutual diffusion process. A consistent trend is evident in both the mutual diffusion system and at the molecular structure level, where rejuvenating agents, being smaller molecules, infiltrate and occupy the voids within asphaltene agglomerations, thus reducing the self-agglomeration of the asphaltene and ultimately enhancing the blending performance, as described in Figure 17. In addition, the steric repulsion between the organic waxes and the side chains of the asphaltene molecules counteracts the π-π interactions between the fused aromatic nuclei of the asphaltene molecules, thus further minimizing the aggregation of RAP asphaltene. In summary, the accelerated diffusion of macromolecules (resins and asphaltenes) and the improved de-aggregation of aged asphaltenes under the dual action of organic wax molecules and rejuvenating agents are the potential reasons for the effective promotion of the blending of the virgin binders with the overall structure of the RAP binders.

## 5. Conclusions

The major contribution of this research is to develop the molecular dynamics model of a warm mix recycled asphalt binder to reveal the thermodynamic performance and microscopic diffusion mechanism changes under the dual action of wax-based additives and oil-based rejuvenators. The glass transition temperature and apparent viscosity were calculated and tested to verify the correctness of the asphalt molecule modeling. The cohesive energy density (CED), self-healing potential, fraction-free volume (FFV), and diffusion coefficient were computed to evaluate the difference in thermodynamic properties and diffusion behavior. The main conclusions can be summarized as follows:

1. The RAP + virgin + wax asphalt system manifested the highest glass transition temperature and apparent viscosity among the three asphalt molecule systems, which were markedly decreased under the dual action of organic waxes and rejuvenators. The simulated glass transition temperature and apparent viscosity keep good consistency with the experimental values, illustrating the feasibility of the asphalt molecule simulations in this study;
2. The presence of RAP binders and organic waxes slightly increased the density, CED, and solubility parameters (δ, δ_vdw_, and δ_ele_) while highly decreasing the surface free energy (SFE), resulting in an enhanced stiffness and poor cracking resistance. The incorporation of rejuvenators was beneficial to revise these thermal properties to improve the performance of the asphalt binders;
3. The different asphalt binders had different self-healing times, and the self-healing performance of the Aged + virgin + wax asphalt binder was poorer than the other two asphalt binders. The incorporation of aromatic oils into the Aged + virgin + wax asphalt binder enhances its self-healing capabilities, as indicated by the earlier attainment of density stabilization, from 450 ps to 375 ps;
4. The free volume of the asphalt binder with organic waxes but without rejuvenators slightly decreased (from 22.02% to 21.9%) in comparison to the asphalt binder with organic waxes and rejuvenators, leading to a greater volume of van der Waals and a decrease in the gaps between unoccupied molecules. And this fact was alleviated after adding a proper dosage of light aromatic oils;
5. The blending process of the virgin and RAP binders was fundamentally achieved through the diffusion of virgin binders. Under the concurrent presence of organic waxes and rejuvenators, the diffusion of macromolecules (resins and asphaltenes) was accelerated, and the de-aggregation of aged asphaltenes was improved, which are the critical factors in effectively promoting the blending of virgin binders with the overall structure of RAP binders.

## 6. On-Going Work

This study discusses the thermodynamic performance and microscopic diffusion mechanism changes in warm mix recycled asphalt with a 50% RAP binder content at the molecular level. In the future, the asphalt molecule models of warm mix recycled asphalt with different RAP binder contents, for example, 20%, 40%, and 60%, will be further investigated. In addition, the asphalt molecule models of warm mix recycled SBS-modified asphalt are expected to be designed with the aim of exploring the presence of SBS polymers on microscopic diffusion behavior.

## Figures and Tables

**Table 2:** Basial properties of the aromatic oil.

Basic Property	Value	Reference Standard
Density	0.890 ± 0.006 g/cm^3^	ASTM D891-2018
Appearance	Dark, viscous liquid	/
Saturated hydrocarbon content	>10%	ASTM D5580-2015
Flash point	170–200 °C	ASTM D92-2018
Boiling point	150–200 °C	ASTM D2887-2022
Aniline point	36 °C	ASTM D611-2016
Engler viscosity (60 °C)	12–15 °E	ASTM D1665-2020

**Table 3:** Comparisons of the simulated and experimental viscosity at different temperatures.

Temperature/°C	Aged and Oil + Virgin + Wax		Aged + Virgin + Wax		Aged and Oil + Virgin	
	Simulated	Experimental	Simulated	Experimental	Simulated	Experimental
115	1426.69	1510	2077.51	2180	1543.91	1490
135	343.32	350	461.73	480	365.43	360
155	109.71	120	139.42	150	115.40	130
175	43.11	50	52.53	60	44.95	50

**Table 4:** Densities, CED, and SFE of the three asphalt binders at the end of MD simulations.

Evaluation Indexes	Aged and Oil + Virgin + Wax	Aged + Virgin + Wax	Aged and Oil + Virgin	Experimental Values
Density (433.15 K, g/cm^3^)	0.9690	0.9893	0.9673	0.95–1.08 at 298.15 K
*CED* (10^8^ J/m^3^)	3.074	3.162	3.045	2.80–3.32
*δ* ((J/cm^3^)^1/2^)	17.53	17.79	17.45	13.30–22.50
*δ_vdw_* ((J/cm^3^)^1/2^)	17.09	17.33	16.71	/
*δ_ele_* ((J/cm^3^)^1/2^)	3.27	3.32	3.18	/
*SFE* (mJ/m^2^)	36.4	18.5	38.2	13.0–47.6

**Table 5:** FFVs of the three asphalt binders.

Binder Types	Occupied Volume/Å^3^	Free Volume/Å^3^	FFV (%)
Aged and oil + virgin + wax	96,275.24	27,193.11	22.02
Aged + virgin + wax	88,956.98	25,034.61	21.96
Aged and oil + virgin	92,411.38	24,603.08	21.03

## Data Availability

The data presented in this study are available in the article.
